# Anosmia and Upper Limb Rigidity—A Potential Phenotype of Idiopathic Normal Pressure Hydrocephalus with Cerebrospinal Fluid α‐Synuclein Seeds

**DOI:** 10.1002/mds.30184

**Published:** 2025-04-09

**Authors:** Sandrina Weber, Carly M. Farris, Yihua Ma, Mohammed Dakna, Maritta Starke, Sebastian Schade, Michael Bartl, Claudia Trenkwalder, Luis Concha‐Marambio, Brit Mollenhauer

**Affiliations:** ^1^ Department of Neurology University Medical Center Goettingen Goettingen Germany; ^2^ Paracelsus‐Elena‐Klinik Kassel Germany; ^3^ R&D Unit Amprion San Diego California USA; ^4^ Institute for Neuroimmunology and Multiple Sclerosis Research, University Medical Center Goettingen Goettingen Germany; ^5^ Department of Neurosurgery University Medical Center Goettingen Goettingen Germany

**Keywords:** anosmia, α‐synuclein, normal pressure hydrocephalus, SAA, seed amplification assay

## Abstract

**Background:**

The pathophysiology of idiopathic normal pressure hydrocephalus (iNPH) and its association with neurodegenerative disorders is poorly understood.

**Objectives:**

The aim was to determine the prevalence of α‐synuclein pathology in iNPH and its associations with clinical characteristics.

**Methods:**

We used α‐synuclein seed amplification assay (synSAA) to retrospectively analyze cerebrospinal fluid (CSF) from a large single‐center iNPH cohort (n = 144). Clinical assessments comprised Unified Parkinson's Disease Rating Scale part III, Mini‐Mental State Examination, levodopa‐challenge test, and olfactory identification test. Degenerative biomarkers (total‐tau, phospho‐tau, β‐amyloid 1–42, and β‐amyloid 1–40) were measured in CSF.

**Results:**

A total of 30.1% of iNPH patients were synSAA+, and presented significantly more upper limb (UL) rigidity, hallucinations, and worse olfactory performance than synSAA− cases. Anosmia was higher in synSAA+ patients (64.0%) than synSAA− patients (15.3%). Clinical assessments and other biomarkers did not significantly vary with synSAA status.

**Conclusions:**

Underlying α‐synuclein pathology is common in iNPH and presents with UL rigidity and olfactory dysfunction, suggesting a distinct synSAA+ phenotype in iNPH. © 2025 The Author(s). *Movement Disorders* published by Wiley Periodicals LLC on behalf of International Parkinson and Movement Disorder Society.

In the field of movement disorders, there is probably no diagnosis that is as common and at the same time as under investigated and controversial as idiopathic normal pressure hydrocephalus (iNPH).^1^ Defining features are ventriculomegaly and gait disturbance, combined with cognitive impairment and/or urinary symptoms (incontinency/urgency).^2^ The prevalence of iNPH is high, and estimated to be approximately 0.4% to 1.5% in the population over 65 years.^3^, ^4^ Because of ageing society, the number of iNPH patients and the associated morbidity and mortality will increase in the upcoming years. Diagnosis of iNPH is challenging, because symptoms are rather unspecific and common in the elderly population. Furthermore, universally accepted diagnostic guidelines are lacking,^1^ and the diagnosis relies on symptomatic improvement of gait or cognition after cerebrospinal fluid (CSF) removal, which has low sensitivity and specificity in predicting response to surgical treatment.^5^ Drainage of CSF via shunt surgery is the gold‐standard treatment and can potentially reverse clinical symptoms, if applied early. However, the disease still progresses afterward in a considerable number of patients.^5^ This variable treatment response, and the observation that neurodegenerative disorders are eventually diagnosed in more than 25% of patients, raises questions about the proportion of iNPH that is truly idiopathic and how many cases are rather clinical presentations of an underlying neurodegeneration.^1^, ^5^ Another possibility is that impaired glymphatic function, which is seen in ventriculomegaly, can lead to impaired clearance of proteins and increased protein‐aggregation, thereby possibly triggering a secondary neurodegeneration.^6^, ^7^ Therefore, a better understanding of the etiology of iNPH is essential for improving diagnosis, treatment, and management.

Until recently, progress in understanding the etiology of iNPH has been hindered by the lack of reliable and readily available tools to assess underlying neurodegenerative processes. The introduction of the α‐synuclein seed amplification assay (synSAA) marks an important advancement that enables the identification of underlying α‐synuclein pathology by detecting misfolded α‐synuclein (syn‐seeds) in CSF with high sensitivity and specificity.^8^, ^9^, ^10^ This advancement provides an opportunity to deepen our understanding of iNPH's potential relationship with α‐synuclein aggregation diseases. Furthermore, a diagnostic approach that incorporates biomarkers and stratifies patients according to underlying neuropathology could eventually improve diagnosis and treatment. However, only few studies have analyzed the presence of syn‐seeds in iNPH to date.^11^, ^12^

Consequently, the objective of this study was to assess the presence of syn‐seeds in the CSF of a large iNPH cohort using synSAA and to explore possible associations with clinical characteristics.

## Materials and Methods

### Study Participants

Patients were recruited at the specialized Parkinson's and movement disorder center, Paracelsus‐Elena‐Klinik, Kassel, Germany. The study was approved by the local ethics committee and all participants signed an informed consent. Diagnosis of iNPH, according to established criteria,^2^ was made by experienced movement disorder neurologists (Supporting Information S1). Patients with the diagnosis of Parkinson's disease (PD)^13^ or dementia with Lewy‐Bodies (DLB)^14^ according to diagnostic criteria were excluded. Clinical examinations included assessment of motor symptoms with the Unified Parkinson's Disease Rating Scale part III (UPDRS‐III) and cognitive function with the Mini‐Mental State Examination (MMSE).^15^ Levodopa‐response was determined by levodopa‐challenge test as described previously,^16^ with a greater than 30% improvement in UPDRS‐III defined as positive result. Medical history interviews, basic psychiatric assessment, and video polysomnography (vPSG) results were reviewed for information on hallucinations and rapid eye movement sleep behavior disorder (RBD) (Supporting Information S1). Olfaction was assessed with the abbreviated 12 item “Sniffin’ Sticks”^17^ test for smell identification. Olfactory performance was classified according to correctly identified smells (≥10 normosmia, 9–7 hyposmia, ≤6 anosmia).

### CSF Biomarkers

CSF collection, processing, and quantification of β‐amyloid 1–42, β‐amyloid 1–40, total and phosphorylated tau protein have been described previously.^18^, ^19^ The qualitative synSAA was performed by Amprion, and the type of syn‐seeds (type 1, associated with Lewy‐Bodies [LB] in PD, DLB, and type 2, associated with glial cytoplasmic inclusions [GCI] in multisystem atrophy [MSA]) was determined as previously described (Supporting Information S1).^10^

### Statistical Analysis

Statistical analysis was performed using R software (version 4.4.1). Group comparisons were made with the non‐parametric Wilcoxon‐test because of some non‐normal distributions. Fischer's exact test was used for nominal variables with two categories or binary ones, and the χ^2^ test for variables with more than two categories. Multiple comparisons were corrected by controlling the false discovery rate using the Benjamini‐Hochberg procedure.^20^

### Data Sharing

All relevant data are within the manuscript and the Supporting Data S1. Additional data is available on request.

## Results

**FIG. 1:**
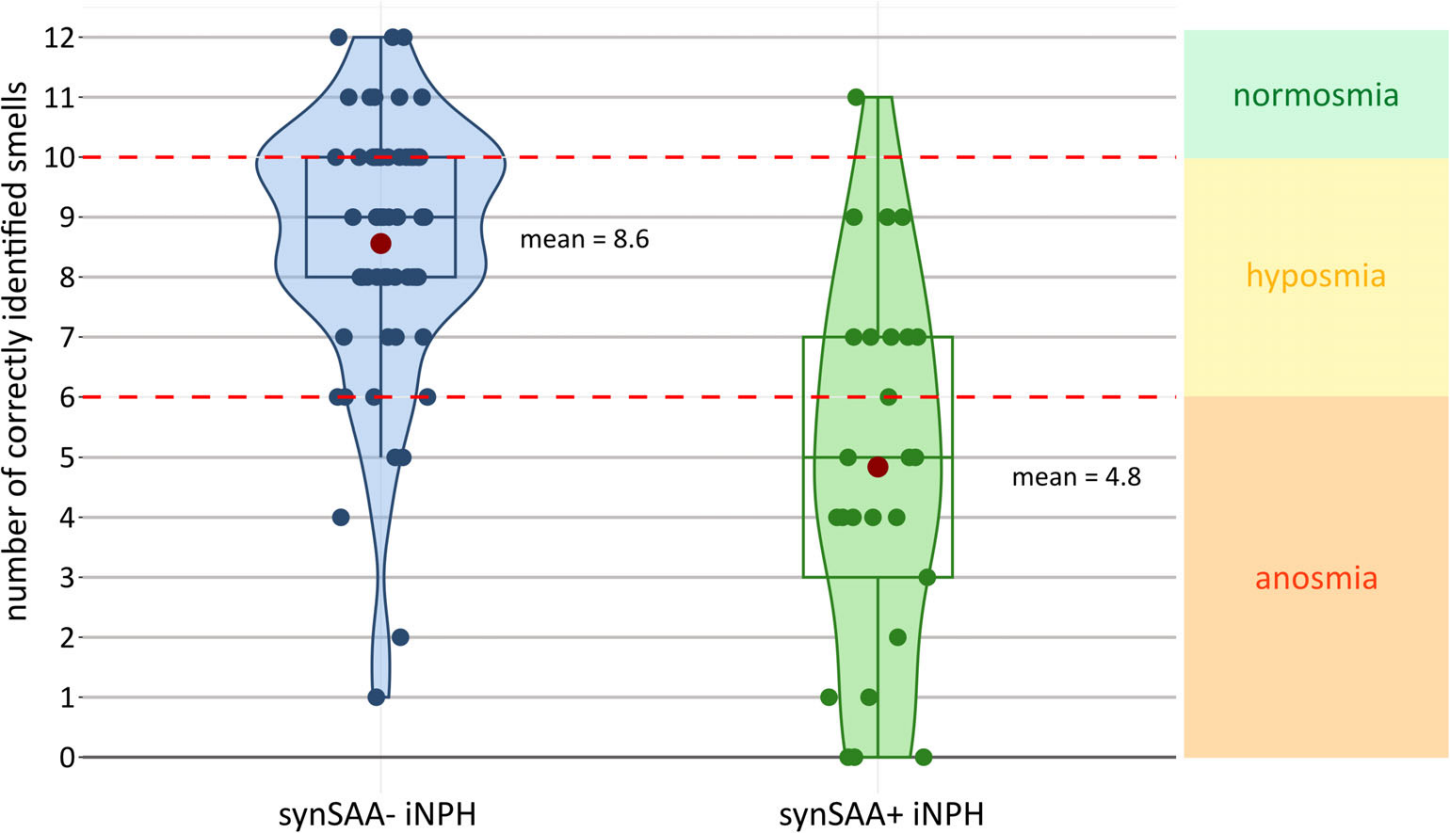
Violin plots showing the number of correctly identified smells (maximum 12) in the “Sniffin’ Sticks” olfactory identification test of α‐synuclein seed amplification assay negative (synSAA‐) and α‐synuclein seed amplification assay positive (synSAA+) idiopathic normal pressure hydrocephalus (iNPH) patients. Olfactory performance: ≥10 normosmia, 9–7 hyposmia, ≤6 anosmia. [Color figure can be viewed at wileyonlinelibrary.com]

Of 167 recruited normal pressure hydrocephalus (NPH) patients, 23 had a concomitant diagnosis of PD and were excluded. Of the remaining 144 iNPH, 143 had a conclusive synSAA result, one iNPH was synSAA inconclusive and excluded from further analyses. In total, 43 of 143 (30.1%) samples were synSAA positive (synSAA+), and 100 of 143 (69.9%) synSAA negative (synSAA−). All syn‐seeds were type 1, associated with LB (as opposed to type 2, associated with MSA).^10^ Sex distribution, mean age at sampling, mean disease duration, and frequency of dopaminergic treatment were not significantly different between synSAA− and synSAA+ (*P* > 0.05; Table 1). The combination of gait disorder and cognitive impairment was slightly more common in synSAA+ than synSAA− patients (95.3% vs. 83.0%), as well as the full triad (gait disorder, cognitive impairment, and urinary symptoms) (74.4% synSAA+ vs. 61.0% synSAA−). SynSAA+ patients had a slightly higher mean UPDRS‐III score (25.9) and lower mean MMSE score (23.0) than synSAA− patients (mean UPDRS‐III 24.2; mean MMSE 24.6). To assess clinical signs associated with PD, we compared UPDRS‐III subscores for speech, facial expression, rigidity of neck, and upper limbs (UL), rest tremor of face, rest tremor of UL, and lower limbs (LL), and hypokinesia of UL. With the exception of facial rest tremor, all other average subscores were higher in synSAA+, with a statistically significant difference for UL rigidity (*P* = 0.017). Positive levodopa‐response was more common in synSAA− (5.3% vs. 0%). Data on RBD was available for a subgroup of patients (35 synSAA−, 16 synSAA+; in total 8 results from vPSG) and RBD was more common in synSAA+ (43.8% vs. 17.1%), without reaching statistical significance. Hallucinations were significantly more common in synSAA+ than synSAA− (23.3% vs. 6%; *P* = 0.017). SynSAA+ participants performed significantly worse at olfactory testing regarding the average number of identified smells (4.8 vs. 8.6; *P* < 0.001). Only 15.3% of synSAA− were anosmic, compared to 64.0% of synSAA+ (Fig. 1). Although there was overlap in the hyposmia category (44.1% synSAA−; 32.0% synSAA+), normosmia was 10 times more common in synSAA− (40.7% vs. 4.0%). There were no significant differences between groups regarding CSF biomarkers (Table 1). Because olfactory testing was only available in 84 patients (58.7%), we compared clinical features within this subgroup. There was also no statistical difference in sex, age at sampling, disease duration, dopaminergic treatment, and levodopa‐response. Cognitive impairment was more common in synSAA+ (92.0% vs. 78.0%) and MMSE significantly lower (23.6 vs. 25.9, *P* = 0.049). UPDRS‐III subscores were higher in synSAA+, except for speech, facial rest tremor, and UL hypokinesia. A significant difference was also observed for UL rigidity. RBD was more common in synSAA+ (55.6% vs. 24.0%). There was no significant difference between CSF biomarkers. We examined whether impaired olfaction in synSAA− iNPH is linked to increased tau, suggesting Alzheimer's pathology. Although total‐tau and phospho‐tau levels were slightly higher in anosmic/hyposmic patients, the difference was not statistically significant (Supplementary Table S1).

**TABLE 1:** Clinical characteristics in synSAA− and synSAA+ iNPH.

	synSAA− iNPH (n = 100)	synSAA+ iNPH (n = 43)	*P*‐value*
n	100 (69.9%)	43 (30.1%)	
Demographics			
Female	29 (29.0%)	13 (30.2%)	0.922
Male	71 (71.0%)	30 (69.8%)	
Age at sampling (y)	76.2 (5.8)	77.4 (5.3)	0.493
Duration of disease (y)	2.9 (1.8)	3.1 (2.0)	0.922
Clinical characteristics			
Gait disorder + urinary symptoms	78 (78.0%)	34 (79.1%)	0.922
Gait disorder + cognitive impairment	83 (83.0%)	41 (95.3%)	0.150
Gait disorder + urinary symptoms and cognitive impairment	61 (61.0%)	32 (74.4%)	0.290
Dopaminergic treatment^a^	47 (47.0%)	21 (48.8%)	0.922
MMSE	24.6 (4.6)	23.0 (5.2)	0.152
Missing values	4	4	
Levodopa‐response^b^	4 (5.3%)	0 (0.0%)	0.467
Missing values	24	15	
RBD^c^	6 (17.1%)	7 (43.8%)	0.150
Missing values	65	27	
Hallucinations	6 (6.0%)	10 (23.3%)	**0.017**
UPDRS‐III			
Total score	24.2 (12.2)	25.9 (11.9)	0.570
Speech	0.9 (0.8)	1.0 (0.7)	0.493
Facial expression	0.9 (0.7)	1.3 (0.8)	0.086
Rest tremor face	0.0 (0.1)	0.0 (0.0)	0.696
Rest tremor UL	0.2 (0.7)	0.4 (0.9)	0.629
Rest tremor LL	0.0 (0.2)	0.2 (0.6)	0.257
Rigidity of neck	0.5 (0.8)	0.9 (0.8)	0.055
Rigidity UL	1.0 (1.4)	1.9 (1.5)	**0.017**
Hypokinesia UL	4.5 (3.6)	4.9 (3.5)	0.687
Missing values	9	8	
Olfactory performance			
Average n of correctly identified smells	8.6 (2.2)	4.8 (3.1)	**<0.001**
Anosmic patients (score ≤6)	9 (15.3%)	16 (64.0%)	**<0.001**
Hyposmic patients (score 7–9)	26 (44.1%)	8 (32.0%)	
Normosmic patients (score ≥10)	24 (40.7%)	1 (4.0%)	
Missing values	41	18	
CSF biomarkers			
Total‐tau (pg/mL)	184.7 (111.5)	169.8 (88.1)	0.822
Missing values	2	3	
Phospho‐tau (pg/mL)	33.9 (17.1)	35.0 (12.7)	0.570
Missing values	30	12	
Aβ1‐42 (pg/mL)	635.0 (212.9)	595.9 (253.8)	0.523
Missing values	2	3	
Aβ1‐40 (pg/mL)	6510.5 (3254.1)	6195.0 (2298.9)	0.963
Missing values	27	12	

Subgroup of iNPH with olfactory testing	synSAA− iNPH (n = 59)	synSAA+ iNPH (n = 25)	*P*‐value*
Demographics			
Female	17 (28.8%)	5 (20.0%)	0.625
Male	42 (71.2%)	20 (80.0%)	
Age at sampling (y)	75.6 (6.1)	76.9 (5.5)	0.625
Duration of disease (y)	2.9 (1.7)	3.3 (2.0)	0.625
Clinical characteristics			
Gait disorder + urinary symptoms	47 (79.7%)	20 (80.0%)	0.972
Gait disorder + cognitive impairment	46 (78.0%)	23 (92.0%)	0.360
Gait disorder + urinary symptoms and cognitive impairment	34 (57.6%)	18 (72.0%)	0.478
Dopaminergic treatment^a^	32 (54.2%)	15 (60.0%)	0.741
MMSE	25.9 (3.5)	23.6 (4.2)	**0.049**
Missing values	2	1	
Levodopa‐response^b^	2 (4.1%)	0 (0.0%)	0.625
Missing values	10	7	
RBD^c^	6 (24.0%)	5 (55.6%)	0.294
Missing values	34	16	
Hallucinations	3 (5.1%)	7 (28.0%)	**0.026**
UPDRS‐III			
Total score	24.3 (11.5)	26.5 (13.2)	0.714
Speech	1.0 (0.7)	1.0 (0.7)	0.741
Facial expression	1.1 (0.7)	1.4 (0.8)	0.294
Rest tremor face	0.0 (0.1)	0.0 (0.0)	0.714
Rest tremor UL	0.3 (0.8)	0.5 (1.1)	0.539
Rest tremor LL	0.0 (0.0)	0.2 (0.7)	0.109
Rigidity of neck	0.6 (0.9)	0.9 (0.8)	0.478
Rigidity UL	1.2 (1.4)	2.1 (1.5)	**0.042**
Hypokinesia UL	5.1 (3.6)	4.8 (3.8)	0.878
Missing values	2	2	
Olfactory performance			
Average no. of correctly identified smells	8.6 (2.2)	4.8 (3.1)	**<0.001**
Anosmic patients(score ≤6)	9 (15.3%)	16 (64.0%)	**<0.001**
Hyposmic patients(score 7–9)	26 (44.1%)	8 (32.0%)	
Normosmic patients (score ≥10)	24 (40.7%)	1 (4.0%)	
Missing values	0	0	
CSF biomarkers			
Total tau (pg/mL)	183.4 (114.9)	173.0 (92.4)	0.972
Missing values	0	1	
Phospho‐tau (pg/mL)	36.1 (19.1)	33.7 (12.2)	0.972
Missing values	15	6	
Aβ1‐42 (pg/mL)	669.0 (222.3)	587.4 (288.9)	0.478
Missing values	0	1	
Aβ1‐40 (pg/mL)	7015.2 (3735.4)	6029.8 (2516.1)	0.681
Missing values	11	7	

*Note*: Values are n (%) or mean (standard deviation) if not otherwise specified. Statistically significant different values are bold.

Abbreviations: synSAA−, α‐synuclein seed amplification assay negative; synSAA+, α‐synuclein seed amplification assay positive; iNPH, idiopathic normal pressure hydrocephalus; MMSE, Mini‐Mental State Examination; RBD, rapid eye movement sleep behavior disorder; UPDRS‐III, Unified Parkinson's Disease Rating Scale part III; UL, upper limbs; LL, lower limbs; CSF, cerebrospinal fluid; Aβ, β‐amyloid.

^a^ Dopaminergic treatment (levodopa and/or dopamine agonists).

^b^ Positive response defined as >30% improvement in UPDRS‐III.

^c^ According to medical history interviews or results of video polysomnography.

* All *P*‐values were adjusted for multiple comparisons using the Benjamini‐Hochberg procedure.

## Discussion

The idiopathic nature of iNPH has been increasingly challenged in recent years.^1^ Some experts suggest the concept of neurodegenerative NPH, where ventriculomegaly and gait, cognitive, or urinary symptoms may be caused by an underlying neurodegenerative process.^5^ Alternatively, neurodegenerative markers found in iNPH may simply represent age‐related incidental pathology rather than a causal factor.

We detected syn‐seeds with synSAA in 30.1% of iNPH patients. This exceeds the frequency of incidental α‐synuclein pathology^21^, ^22^, ^23^ and syn‐seeds^24^ (detected by synSAA) in aged controls and therefore, supports an association between iNPH and underlying α‐synuclein pathology, even without clinical PD. Previous studies found syn‐seeds in 14.0% and 20.5% of iNPH patients, respectively.^11^, ^12^ The higher positivity in our study could be a result of increased synSAA sensitivity^10^ or patient selection, because our cohort included patients that had been referred to a movement disorder center for diagnostic evaluation, often with the question whether a patient had iNPH or PD. It is, therefore, possible that our iNPH cohort was enriched for patients with clinical motor signs suggestive of PD, although not meeting diagnostic criteria for PD. In this regard, certain parkinsonian motor symptoms in iNPH have been associated with a higher rate of synSAA positivity before.^12^

Concerning characteristic PD motor features, our analysis showed that synSAA+ patients had significantly more UL rigidity, thereby reproducing the finding from a previous study^12^ in an independent cohort, underscoring UL rigidity as a potential characteristic of synSAA+ iNPHs. However, in contrast to results from previous studies,^11^, ^12^ a positive response to levodopa, judged by an improvement of UPDRS‐III greater than 30%, was not associated with syn‐seeds and there was no difference between groups concerning medication with dopaminergic drugs. Assessing further clinical symptoms associated with synucleinopathies, we found RBD to be more common in synSAA+ patients, contrary to a previous report.^11^ However, this difference was not statistically significant, as it was based on a small subgroup of patients with available data. In addition, it relied primarily on self‐reported RBD symptoms by the patient and/or bed partner, which are often inaccurate and far less reliable than a vPSG‐confirmed diagnosis. Given this limitation, the finding should be interpreted with caution and future studies with systematic vPSG assessments are needed to clarify the relationship between RBD and synSAA+ in iNPH. Interestingly, hallucinations, a hallmark feature of DLB, were significantly more frequent in synSAA+ than synSAA− iNPH patients. To our knowledge, this association has not been reported previously. Although the difference reached statistical significance, the overall number of patients experiencing hallucinations was small, warranting cautious interpretation.

Last, we found that synSAA+ iNPHs had significantly worse olfactory performance than synSAA−, with a higher prevalence of anosmia. Impaired olfaction is a typical non‐motor symptom of PD and usually precedes motor symptoms for several years, suggesting that it is an early clinical indicator of α‐synuclein pathology. Moreover, hyposmia was the most robust predictor of synSAA+ in a large PD and prodromal PD cohort.^8^ Therefore, our findings suggest that olfactory testing could serve as a potential screening tool for identifying α‐synuclein pathology in iNPH patients. Because olfactory testing was available for only approximately half of the study cohort, we conducted a subgroup analysis on these patients to further investigate potential differences between synSAA+ and synSAA− iNPH. Overall, the results were consistent with the findings from the entire cohort, and a significant difference in hallucinations and UL rigidity was also observed within this subgroup. Notably, synSAA+ patients had significantly lower mean MMSE scores, a difference not observed in the full cohort. Future studies with detailed cognitive assessments could explore whether specific cognitive domains are more affected by syn‐seeds than others. For example, a recent study found that Alzheimer's patients with α‐synuclein co‐pathology had significantly greater visuospatial impairment and experienced a more pronounced cognitive decline at follow‐up, providing evidence that syn‐seeds contribute to distinct clinical manifestations.^25^ Investigating whether similar patterns appear in iNPH could help to resolve the important question to which extend α‐synuclein pathology influences specific clinical features in the absence of a synucleinopathy diagnosis.

Our study has some limitations, mainly the cross‐sectionally retrospective study‐design and lack of systematic clinical follow‐up. Additionally, differences between synSAA+/synSAA– regarding response to spinal‐tap or shunt‐surgery and a possible impact of neurovascular pathology on clinical symptoms were beyond the scope of this study. Strengths of this study are the large single‐center cohort with standardized protocols for levodopa‐challenge and olfactory testing and the use of a highly accurate and novel synSAA, which can reliably distinguish syn‐seeds associated with LB pathology and MSA.

In conclusion, we found underlying α‐synuclein aggregation in 30.1% of iNPH patients, which exceeds the frequency of incidental syn‐seeds, and provides evidence for a potential relationship. Clinical PD characteristics, most notably upper limb rigidity and anosmia, were associated with syn‐seeds and support the existence of a distinct synSAA+ iNPH phenotype. Incorporating biomarker‐based diagnostics, such as synSAA, may offer a more accurate classification in ambiguously clinically defined disorders like iNPH. This stratification based on underlying neuropathology could pave the way for more individualized therapeutic strategies.

## Author Roles

(1) Study: A: Conception, B: Organization, C: Execution. (2) Statistical Analysis: A: Design, B: Execution, C: Review and Critique. (3) Manuscript Preparation: A: Writing First Draft, B: Review and Critique.

S.W.: 1A, 1B, 1C, 2C, 3A, 3B.

C.M.F.: 1B, 1C, 3B.

Y.M.: 1B, 1C, 3B.

M.D.: 2A, 2B, 2C, 3B.

S.S.: 1C, 3B.

M.B.: 1C, 3B.

M.S.: 1C, 3B.

C.T.: 1C, 3B.

L.C.M.: 1B, 1C, 3B.

B.M.: 1A, 1B, 1C, 2C, 3B.

## Financial Disclosures

S.W. has received funding from the University Medical Center Goettingen—UMG Clinician Scientist Program. L.C.M., C.M.F., and Y.M. are employees of Amprion, and hold stock options. L.C.M., C.M.F., and Y.M. are named inventors of several patents and pending patent applications related to the α‐synuclein seed amplification assay (synSAA). These patents and pending applications are either co‐owned by Amprion with UT Health and exclusively licensed by Amprion Inc. or are owned solely by Amprion. All inquiries about the use of synSAA for diagnostic and/or drug development purposes should be directed to L.C.M. M.D. is partly supported by funds from The Michael J. Fox Foundation (MJFF‐24360). S. S. received institutional salaries supported by the EU Horizon 2020 research and innovation program under grant agreement no. 863664 and by The Michael J. Fox Foundation for Parkinson's Research (MJFF‐021923). He is supported by a Parkinson's Progression Markers Initiative (PPMI) Early Stage Investigators Funding Program fellowship of The Michael J. Fox Foundation for Parkinson's Research (MJFF‐022656). M.B. has received funding from the Deutsche Forschungsgemeinschaft (DFG, German Research Foundation) – 413,501,650. M.S. has no disclosures to report. C.T. is on the advisory board of AbbVie, Bial, Boehringer Ingelheim, Convatec, Ono Pharmaceutical, Roche and UCB. C.T. has received honoraria from AbbVie, Alexion, Bial, UCB, Esteve and academic grants from ERA Net and PPMI (The Michael J. Fox Foundation). B.M. is advising Amprion Inc. without honoraria and has received honoraria for consultancy from Roche, Biogen, AbbVie, and Bial. B.M. is a member of the executive steering committee of the PPMI of The Michael J. Fox Foundation for Parkinson's Research and has received research funding from the DFG, European Union (Horizon 2020), Aligning Science Across Parkinson's disease (asap, CRN) and Parkinson's Foundation and The Michael J. Fox Foundation for Parkinson's Research.

## Supporting information


**TABLE S1.** Comparison of CSF total tau and phospho‐tau in synSAA−iNPH with available results from olfactory testing.

**DATA S1.** Supporting Information.

## Data Availability

The data that support the findings of this study are available from the corresponding author upon reasonable request.
