# Supplementary material for: Anosmia and Upper Limb Rigidity—A Potential Phenotype of Idiopathic Normal Pressure Hydrocephalus with Cerebrospinal Fluid α‐Synuclein Seeds
Source: Mov Disord. 2025 Apr 9;40(6):1206–13. doi: 10.1002/mds.30184 (PMC12160991; doi:10.1002/mds.30184)
Supplement: Supplementary file 1 — TABLE S1. Comparison of CSF total tau and phospho‐tau in synSAA−iNPH with available results from olfactory testing. [file MDS-40-1206-s002.docx]

**Supplementary Table I**

Comparison of CSF total tau and phospho-tau in synSAA- iNPH with available results from olfactory testing.

|  | synSAA- normosmia  n = 24 | synSAA- hyposmia/anosmia  n = 35 | p-value |
| --- | --- | --- | --- |
| total tau (pg/ml) | 164.5 (86.7) | 196.3 (130.5) | 0.266 |
| missing values | 0 | 0 |  |
| phospho-tau (pg/ml) | 32.9 (13.0) | 38.4 (22.3) | 0.308 |
| missing values | 6 | 9 |  |

Values are mean (standard deviation).
