# Supplementary material for: Anosmia and Upper Limb Rigidity—A Potential Phenotype of Idiopathic Normal Pressure Hydrocephalus with Cerebrospinal Fluid α‐Synuclein Seeds
Source: Mov Disord. 2025 Apr 9;40(6):1206–13. doi: 10.1002/mds.30184 (PMC12160991; doi:10.1002/mds.30184)
Supplement: Supplementary file 2 — DATA S1. Supporting Information. [file MDS-40-1206-s001.docx]

**Anosmia and Upper Limb Rigidity – A Potential Phenotype of Idiopathic Normal Pressure Hydrocephalus with Cerebrospinal Fluid** **α-Synuclein Seeds**

Sandrina Weber, Carly M. Farris, Yihua Ma, Mohammed Dakna, Maritta Starke, Sebastian Schade, Michael Bartl, Claudia Trenkwalder, Luis Concha-Marambio, Brit Mollenhauer*

**Supplemental Material and Methods.**

*Study Participants – Diagnosis and Clinical assessments*

All patients were enrolled as the ‘Kassel cohort’ during an inpatient diagnostic evaluation at the specialized Parkinson’s and movement disorder center Paracelsus-Elena-Klinik, Kassel, Germany. A detailed medical history was taken for each patient to assess urinary incontinence and/or urgency, cognitive impairment / decline, and to rule out secondary causes of non-obstructive hydrocephalus (eg trauma, subarachnoidal haemorrhage, meningitis). Cognitive impairment was assessed with the Mini-Mental State Examination (MMSE)^1^ in the majority of patients. Medical history interviews and a basic psychiatric assessment at the timepoint of admission to the clinic were reviewed for history of hallucinations. The basic psychiatric assessment was done by movement disorder neurologists with experience in psychiatry. All patients had cranial imaging (MRI or CT scan) suggestive of iNPH that was taken during the inpatient workup or prior to referral to the clinic. All imaging studies were analyzed and interpreted by a neuroradiologist. As part of the diagnostic workup, all patients received a high-volume spinal tap with removal of 30 - 40 mL cerebrospinal fluid (CSF). Gait was assessed before spinal tap, immediately after, and 24 hours later. A diagnosis of *probable* or *possible* iNPH according to established criteria^2^ was made by an experienced movement disorder neurologist. Assessment of CSF opening pressure was not part of the routine diagnostic workup and only performed in 7 cases, thus a formal diagnosis of *probable* iNPH according to established criteria (which required an opening pressure within 70 –245 mm H2O) was only possible in 7 cases.

The levodopa-response was assessed with a standardized levodopa-challenge test, as described previously.^3^ For the levodopa-challenge test, the UPDRS-III score was determined after cessation of dopaminergic medication for at least 12 hours overnight. Subsequently, the patient took 250 mg of dispersible levodopa (Madopar LT, Roche Pharma AG, Basel, Switzerland) under fasting conditions and after pre-treatment with domperidone 20 mg (one hour before administration of levodopa, optionally also 12h prior to administration of levodopa). UPDRS-III was determined again 1 h after treatment and the levodopa-response calculated as % of the total UPDRS-III score improvement.

Medical history interviews or results of video polysomnography (vPSG) were reviewed for information on rapid eye movement sleep behavior disorder (RBD). RBD was determined if the history was indicating RBD (history of either dream enactment and/or vocalisations during sleep) according to the information given by the patient or the bed partner or if RBD was diagnosed by video polysomnography (vPSG) at our clinic by experienced raters according to established criteria.^4,5^ A patient was classified as “no RBD” if questions concerning dream enactment and/or vocalisation during sleep were negated during the medical history interview or if vPSG did not show evidence of RBD.

Olfactory performance was evaluated with commercially available 'Sniffin' Sticks' (Burghart Medizintechnik GmbH, Wedel, Germany)^6^ according to the manufacturer's instructions by trained staff. For smell identification, 12 different smells were presented to the patients in order to identify the correct smell. Four possible answers were given for each of the 12 smells from which the patient had to choose the correct smell. Olfactory performance was classified according to the number of correctly identified smells out of 12: ≥10 normosmia, 9-7 hyposmia, ≤6 anosmia.

*CSF Samples – Collection protocol and measurements*

High-volume (between 30 - 40 mL) spinal tap was performed in the morning under fasting conditions using a traumatic needle. CSF was collected in a polypropylene tube and centrifuged at 3,000 x g for 10 min at room temperature. The supernatant was frozen and stored at -80°C. CSF beta-amyloid 1–42, beta-amyloid 1–40, total tau protein, and phosphorylated tau protein (p-tau181) were measured with commercially available INNOTEST ELISA kits (Fujirebio Europe, Ghent, Belgium) as previously described.^7^ Results with a value below a threshold (<75 pg/ml for total-tau, and <15 pg/ml for phospho-tau) were included in the statistical analysis as 75 pg/ml for total-tau and 15 pg/ml for phopsho-tau.

*α-Synuclein Seed Amplification Assay (synSAA)*

Assay conditions have been reported elsewhere.^8^ The synSAA included a final 100µL reaction mixture consisting of 100mM PIPES pH 6.5, 500mM NaCl, 10µM ThT, 0.1% sarkosyl, 0.3mg/mL recombinant α-synuclein substrate (Amprion, cat# S2020), and 40µL of CSF sample. The assay was performed in clear bottom plates with two 3.2mm Si_3_N_4_ beads per well. Plates were evaluated at 42°C with shaking (orbital, 800rpm) for 1min every 15min. Fluorescence (440nm/490nm) was read each 15m cycle with FLUOstar Omegas calibrated with Atto425 fluorescent dye to ensure reproducible measurements among instruments and runs. Briefly, Atto425 dye was resuspended in 200µL DMSO; concentration was determined by absorbance at 429nm (ε_Atto_=43,000M^-1^cm^-1^). Fluorescence (440nm/490nm) of 3.66µM Atto425 was used as the calibrator and set to 77% of the 260,000 RFU maximum range of each FLUOstar Omega. All samples were blindly analyzed in triplicate at Amprion.

The maximum fluorescence (F_max_) in relative fluorescence units (RFU) measured during synSAA were used for the determination criteria. Samples with three high F_max_ (≥45,000 RFU, deemed Type1) replicates are defined synSAA+ Type1. Samples with two or three intermediate F_max_ (≥3000RFU & <45,000RFU, deemed Type2) replicates are defined synSAA+ Type2. Samples with two or three below-threshold (<3,000 RFU, deemed negative) replicates are defined synSAA-. Samples with two Type1 and one Type2 replicates are defined synSAA+ undetermined. Other cases are defined as inconclusive and undetermined. Undetermined cases were retested if sufficient sample volume remained.

**References:**

1. Folstein, M. F., Folstein, S. E. & McHugh, P. R. “Mini-mental state”. *J. Psychiatr. Res.* **12**, 189–198 (1975).

2. Relkin, N., Marmarou, A., Klinge, P., Bergsneider, M. & Black, P. McL. Diagnosing Idiopathic Normal-pressure Hydrocephalus. *Neurosurgery* **57**, S2-4-S2-16 (2005).

3. Schade, S. *et al.* Acute Levodopa Challenge Test in Patients with de novo Parkinson’s Disease: Data from the DeNoPa Cohort. *Mov. Disord. Clin. Pract.* **4**, 755–762 (2017).

4. *International Classification of Sleep Disorders Diagnostic & Coding Manual*. (American Academy of Sleep Medicine, 2005).

5. *International Classification of Sleep Disorders*. (American Academy of Sleep Medicine, 2014).

6. Hummel, T., Sekinger, B., Wolf, S. R., Pauli, E. & Kobal, G. ‘Sniffin’ Sticks’: Olfactory Performance Assessed by the Combined Testing of Odour Identification, Odor Discrimination and Olfactory Threshold. *Chem. Senses* **22**, 39–52 (1997).

7. Mollenhauer, B. *et al.* Monitoring of 30 marker candidates in early Parkinson disease as progression markers. *Neurology* **87**, 168–177 (2016).

8. Ma, Y. *et al.* Sensitivity and specificity of a seed amplification assay for diagnosis of multiple system atrophy: a multicentre cohort study. *Lancet Neurol*.
